# Supplementary material for: Hippocampal ensembles represent sequential relationships among an extended sequence of nonspatial events
Source: Nat Commun. 2022 Feb 8;13:787. doi: 10.1038/s41467-022-28057-6 (PMC8825855; doi:10.1038/s41467-022-28057-6)
Supplement: Supplementary file 1 — Supplementary Information [file 41467_2022_28057_MOESM1_ESM.pdf]

nature COMMUNICATIONS

SUPPLEMENTARY INFORMATION

Hippocampal ensembles represent sequential relationships among an extended sequence of nonspatial events

Babak Shahbaba[1,2,3], Lingge Li[2,3], Forest Agostinelli[2,3,4], Mansi Saraf[5,6], Keiland W. Cooper[5,6], Derenik Haghverdian[2], Gabriel A. Elias[5,6], Pierre Baldi[2,3], and Norbert J. Fortin[1,5,6 *]

[1] Data Science Institute, University of California, Irvine, CA, USA.

[2] Department of Statistics, University of California, Irvine, CA, USA.

[3] Department of Computer Science, University of California, Irvine, CA, USA.

[4] Artificial Intelligence Institute, University of South Carolina, Columbia, SC.

[5] Center for the Neurobiology of Learning and Memory, University of California, Irvine, CA, USA.

[6] Department of Neurobiology and Behavior, University of California, Irvine, CA, USA.

* Correspondence to: norbert.fortin@uci.edu

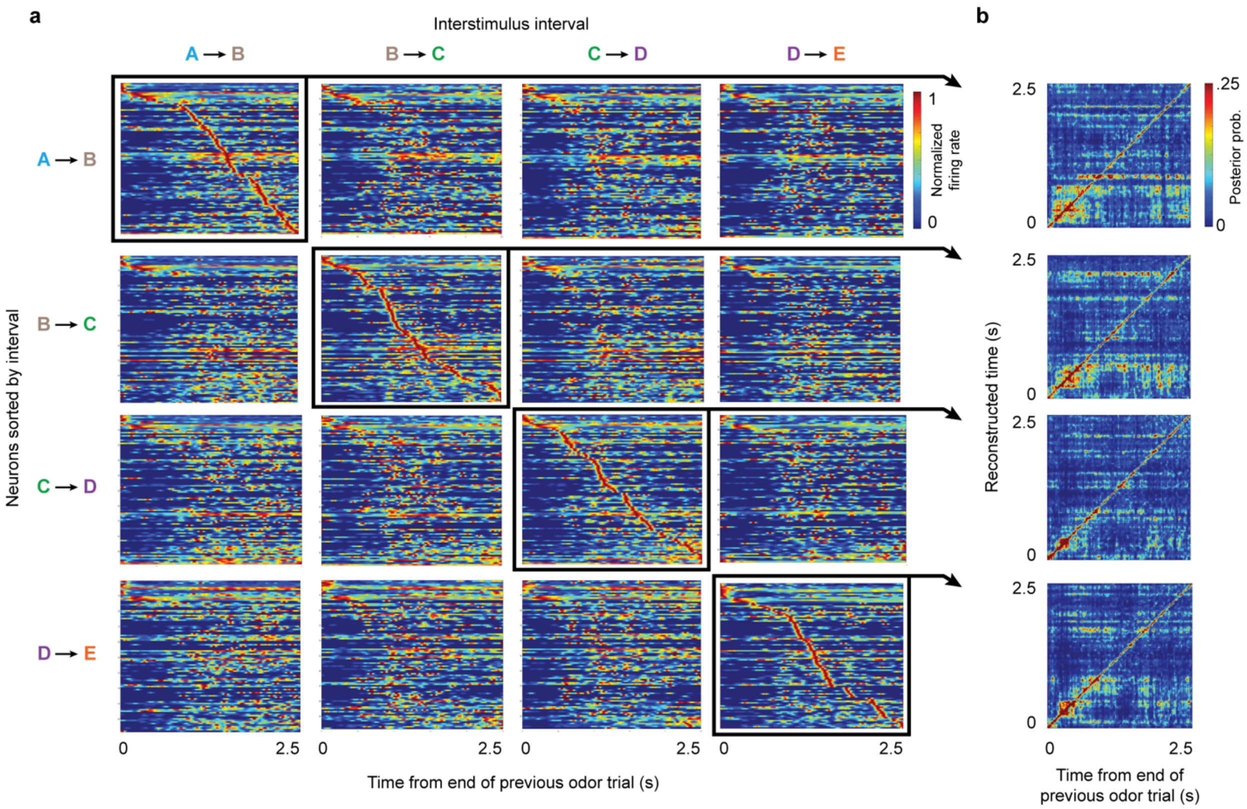

Supplementary Fig. 1 | Representative sequential firing fields ("time fields") during inter-stimulus intervals.

a Ensemble activity from one example subject during inter-odor intervals (0 s corresponds to reward delivery at end of previous odor; plots are terminated at 2.5 s but some intervals were of longer duration). Each peri-stimulus time histogram (PSTH) shows the normalized firing rate of all active neurons during a specific inter-stimulus interval (98 active neurons for each interval, except for B-C interval which had 99 active neurons). To visualize how firing fields varied across interval types, PSTHs are shown for each interval (in columns), with neurons sorted by their time of peak firing relative to the start of each interval (in rows). **b** Temporal coding accuracy for each interval type. Plots show reconstructed time estimates obtained from the example animal's PSTH (correctly sorted intervals; i.e., diagonal of panel **a**). Color coding of odor types: odor A=sky blue, B=brown, C=green, D=purple, E=orange.

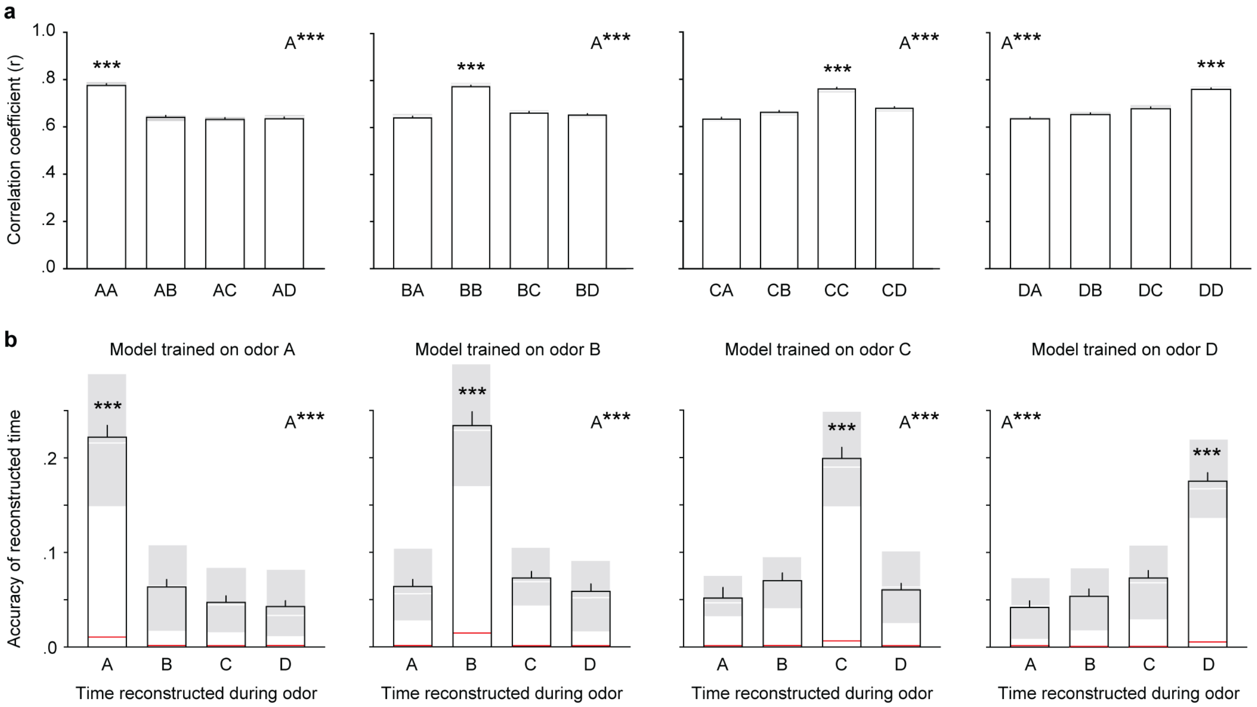

Supplementary Fig. 2 | Stimulus specificity of sequential firing fields.

a Mean correlation coefficient for pairwise comparisons between trial-specific PSTHs (each PSTH including all 370 neurons, collapsed across subjects), categorized by odor types. A one-way ANOVA showed that coefficients of comparisons centered on odor A trials (PSTH of each odor A trial being compared to PSTH of each other A, B, C, or D trial; see top row of Fig. 2a) were significantly different across trial types (left, A vs A,B,C, or D: $F_{(3, 52)} = 336.9$, $p < 0.0001$), with correlations between trials of the same type being significantly higher than the others (AvsA was significantly higher than AvsB, AvsC, or AvsD). The same pattern of results was obtained when the corresponding analyses were performed on comparisons centered on odor B trials (B vs A,B,C, or D: $F_{(3, 52)} = 334.4$, $p < 0.0001$; BvsB significantly higher than others), odor C trials (C vs A,B,C, or D: $F_{(3, 52)} = 258.2$, $p < 0.0001$; CvsC significantly higher than others), and odor D trials (D vs A, B, C, or D: $F_{(3, 52)} = 237.9$, $p < 0.0001$; DvsD significantly higher than others). **b** Analysis of reconstructed time across trial type. A one-way ANOVA showed that when the model was trained using odor A trials, its reconstructed time accuracy significantly varied when applied to odor A, B, C, or D trials ($F_{(3, 216)} = 113.1$, $p < 0.0001$), with the highest accuracy observed when decoding the same trial type as in model training. Again, a similar pattern of results was obtained when the model was trained using odor B trials ($F_{(3, 216)} = 85.33$, $p < 0.0001$, odor C trials ($F_{(3, 216)} = 88.77$, $p < 0.0001$, and odor D trials ($F_{(3, 216)} = 71.27$, $p < 0.0001$). Data depicted as mean ± SEM (n=14 observations per bar in **a**, n=55 observations per bar in **b**), with shaded regions reflecting Q1-Q3 range (median as white line). A***, significant one-way ANOVA (p<0.0001). ***, significant two-tailed Dunnett's posthoc tests (p<0.0001, adjusted for multiple comparisons). Red lines in **b** depict chance levels determined by permutations.

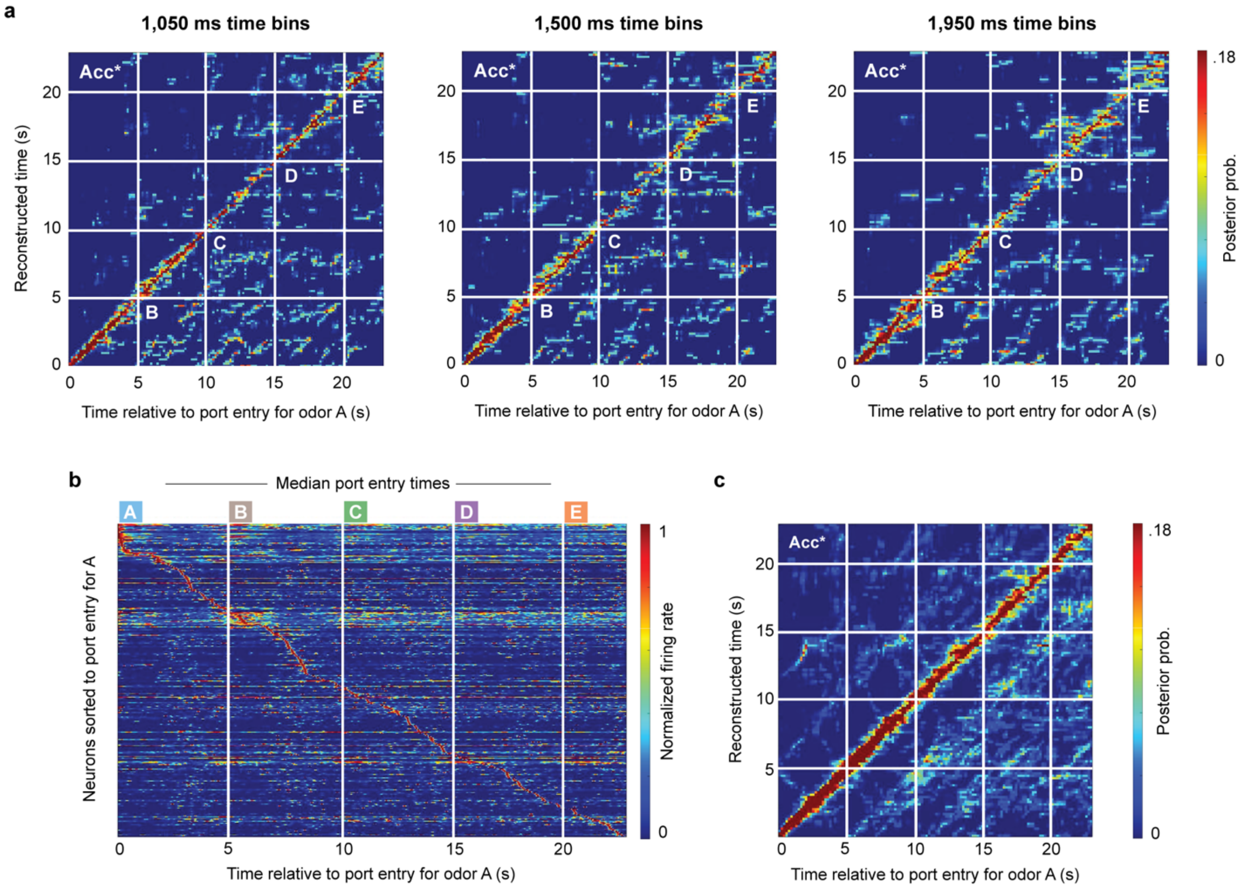

Supplementary Fig. 3 | Temporal coding accuracy across event sequences showed the same pattern when using different time bins and averaging across animals.

a Reconstructed accuracy was comparable using shorter and longer time bins. Data from example subject in Figure 2e are shown in the middle panel for comparison. The same analyses were then performed using shorter time bins (left; 1,050 ms with step size of 150 ms) and longer time bins (right; 1,950 ms, with step size of 150 ms). Reconstructed time accuracy was significantly higher than chance levels using either 1,050 ms time bins (two-tailed one-sample t-test; $t_{(13)} = 9.580$, $p < 0.0001$) or 1,950 ms time bins (two-tailed one-sample t-test; $t_{(13)} = 11.12$, $p < 0.0001$), indicating that in both cases sequential firing fields provided significant temporal information across the full sequence of events (compare with 1,500 ms time bins in middle panel: two-tailed one-sample t-test; $t_{(13)} = 7.941$, $p < 0.0001$). **b**,**c** Data averaged across subjects shows the same temporal organization observed in individual subjects. Even with differences in the timing of odors across subjects (the task is self-paced), averaging across subjects revealed a strong sequential organization of firing fields (**b**; compare with Fig. 2d) and temporal coding (**c**, compare with Fig. 2e) across the full sequence of odors (two-tailed one-sample t-test; $t_{(54)} = 13.95$, $p < 0.0001$). Acc*, reconstructed time accuracy significantly above chance levels (determined by random permutations). Color coding of odor types: odor A=sky blue, B=brown, C=green, D=purple, E=orange.

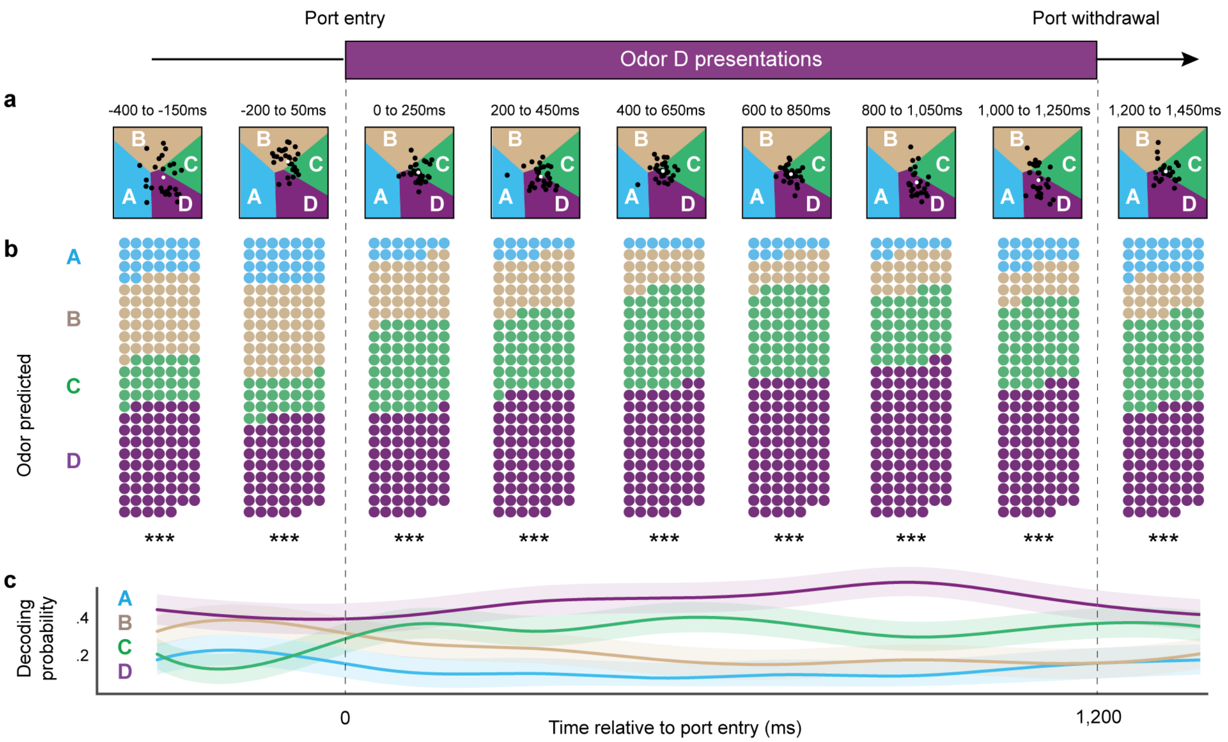

Supplementary Fig. 4 | Reactivation of discontiguous event representations during odor D presentations.

a Decoding dynamics of same example subject as in Figure 4. Regions of latent space are color coded according to the odor of highest probability (odor A=sky blue, B=brown, C=green, D=purple), with boundaries indicating equal probability between two odors. Each black dot indicates the latent representation of a trial in that time window, with its position representing the decoded probabilities for each odor (cluster centroids shown as white circles). **b**,**c** Group decoding dynamics. Since latent space coordinates were specific to each subject's neuronal ensemble, trial data was pooled across subjects according to each trial's highest probability value. Specifically, for each time window, trials (represented by circles; n=165) were categorized and color-coded by their decoded odor of highest probability (**b**). The same data are shown in a summary line plot (mean ± 95% multinomial CI; smoothed using cubic splines) to highlight the decoding probability dynamics within trials (**c**). ***, significant chi-square tests (df=3, p<0.0001).

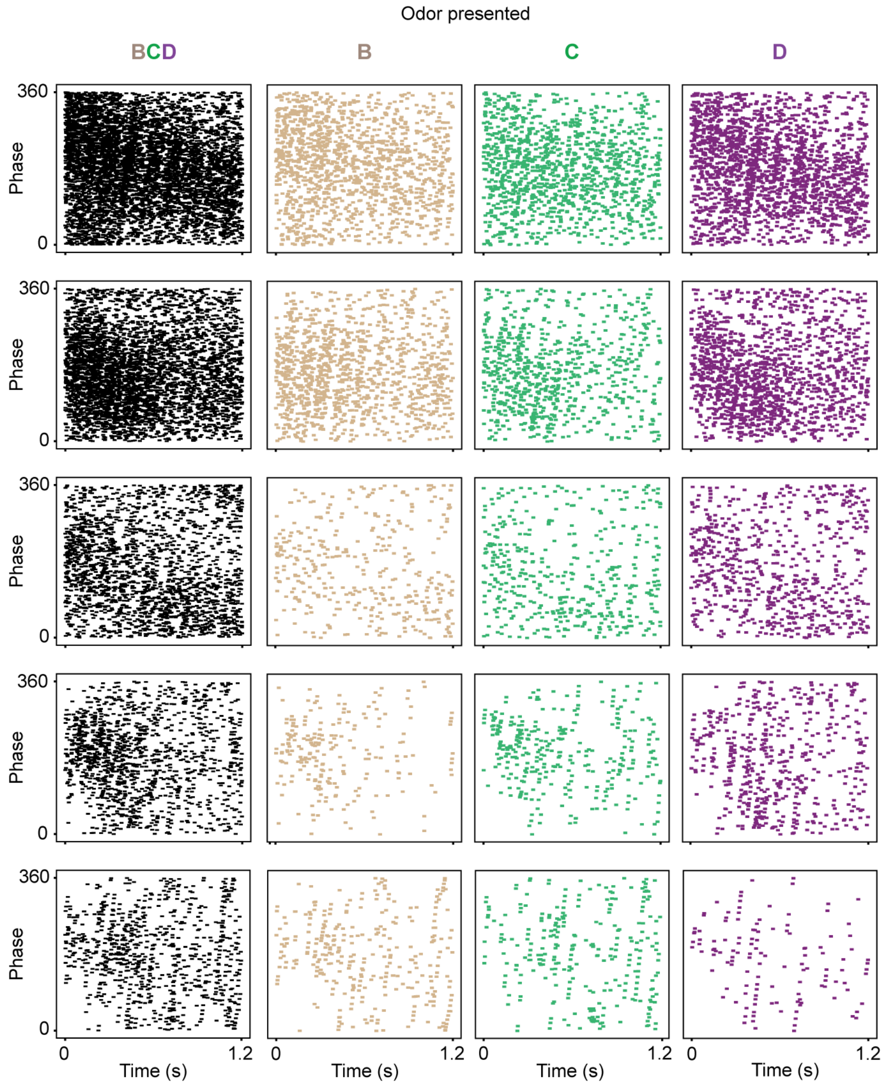

Supplementary Fig. 5 | Examples of theta phase precession at the single-cell level.

Spike rasters from five example neurons (in rows) showing statistically significant theta phase precession during odor presentations. Data shown are for correct InSeq trials organized by presented odors in columns: odor B, C, and D trials combined (column 1; black), odor B trials only (column 2; brown), odor C trials only (column 3; green), and odor D trials only (column 4; purple).

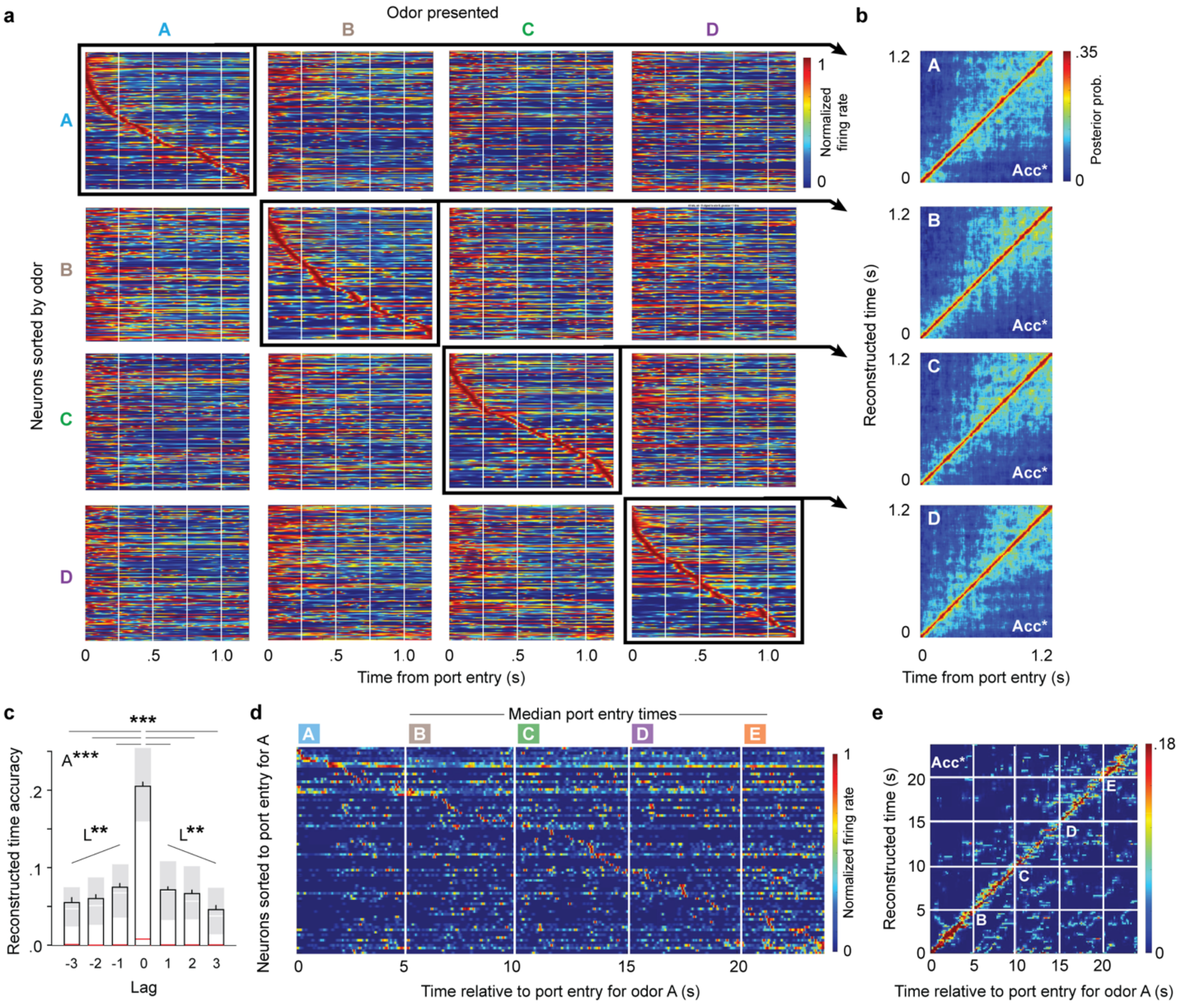

Supplementary Fig. 6 | Temporal coding analyses excluding non-informative neurons reproduce the pattern obtained from the entire ensemble of active neurons.

The same analyses performed on all active neurons in Figure 2 were re-computed using only informative neurons (the 81% that reached statistical significance on the individual neuron temporal reconstruction analysis). **a** Stimulus specificity in sequential firing fields. Peri-stimulus time histograms (PSTH; 150 ms gaussian) show the normalized firing rate of all active neurons for each odor type (correct InSeq trials only; active neurons for each odor: A=225, B=157, C=175, D=181; neurons collapsed across five subjects, one session per subject). PSTHs are shown for each odor presented (in columns), with neurons sorted by their time of peak firing relative to the port entry for each odor (in rows). **b** Temporal coding accuracy for each odor type was above chance levels. Plots show reconstructed time estimates obtained from each animal's PSTH (correctly sorted for each odor; i.e., diagonal of panel **a** but separated by animal) and averaged across subjects. **c** Accuracy of reconstructed time varied by the lag between the odor type used to train the model and the odor type in which time was reconstructed. Black bars depict mean ± SEM (Lag0: *n*=220; Lag±1: *n*=165; Lag±2: *n*=110; Lag±3: *n*=55; trial data pooled across subjects), shaded regions the Q1-Q3 range (median denoted by white line), and red lines the permuted chance levels. Lag data show a significant one-way ANOVA ($F_{(6,873)}$=198.7, p<0.0001; A***), difference between lag 0 and all other lags (two-tailed Dunnett's posthoc tests, adjusted for multiple comparisons; ***, p<0.0001), and linear trends (positive lag: $F_{(1,327)}$=9.536, p=0.0024; negative lag: $F_{(1,327)}$=12.08, p=0.006; L**). **d** The sequential organization of firing fields extended across the full sequence of odors. Example PSTH (250 ms gaussian) of only informative neurons from the same subject as Figure 2 with median port entry times for odors B, C, and D marked across trials. **e** Accuracy of reconstructed time across the sequence of odors (PSTH from panel **d**) was above chance levels. Acc*, reconstructed time accuracy significantly above chance levels (determined by random permutations). Color coding of odor types: odor A=sky blue, B=brown, C=green, D=purple, E=orange.

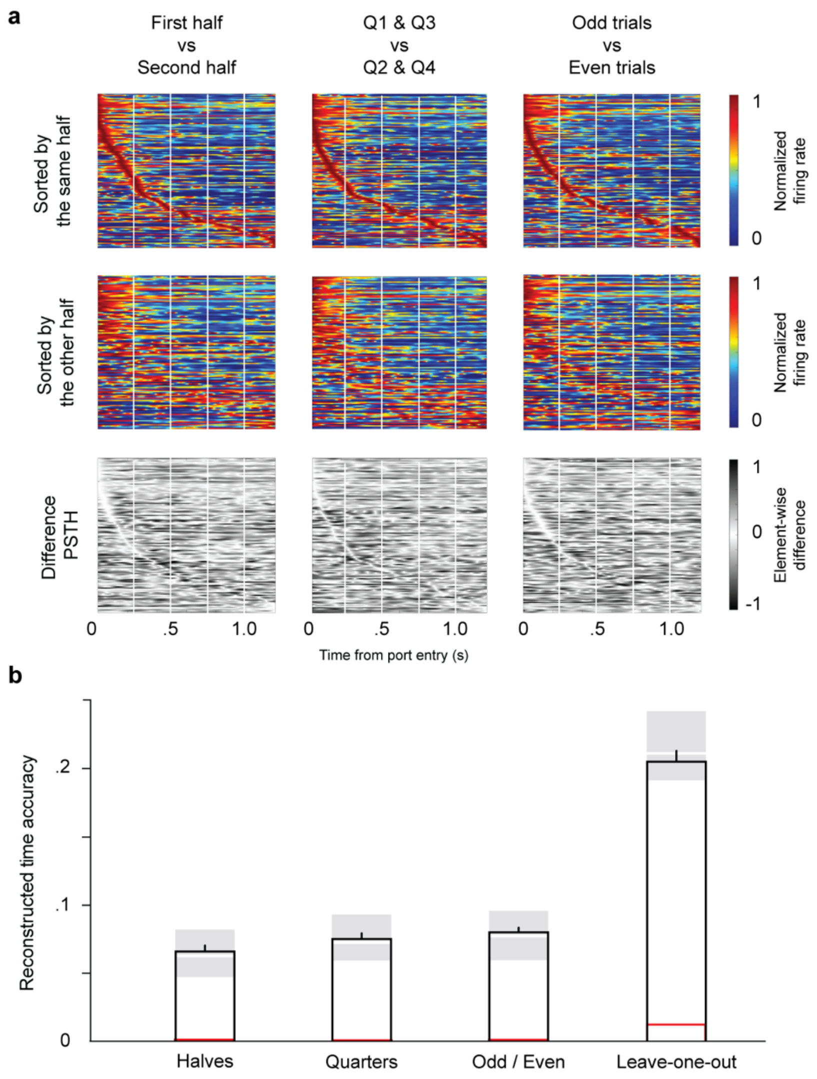

Supplementary Fig. 7 | Temporal coding remains statistically significant using a conservative 50:50 training/test validation approach.

a PSTHs from odor A trials split into different training-testing groupings for decoding (in columns: first vs second half, quarters 1&3 vs quarters 2&4, odd vs even trials). The top row shows normalized firing rate PSTHs for example training sets (first half, Q1&Q3, and odd trials), with neurons sorted by their peak firing latencies. The middle row shows the same data as in the top row, but with neurons sorted by the complementary testing sets (second half, Q2&Q4, even trials). The bottom row shows difference PSTHs (subtracting PSTHs from the two rows above) to visualize the similarity in temporal organization (zero, shown in white, indicates the same activity level between PSTHs). **b** Reconstructed time accuracy on odor A across training-testing groupings, with results from the leave-one-out cross-validation (the main approach used in the paper) shown as a reference. All training-testing splits resulted in reconstructed time accuracy that was significantly above chance (chance determined by random permutations). Bars depict mean ± SEM (halves: n=54 trials; quarters: n=50 trials; odd/even: n=54 trials; leave-one-out: n=55 trials; trial data pooled across subjects), shaded regions the Q1-Q3 range (median denoted by white line), and red lines the permuted chance levels. For clarity, only data from odor A trials are shown but the same pattern was observed with odors B, C, and D trials.

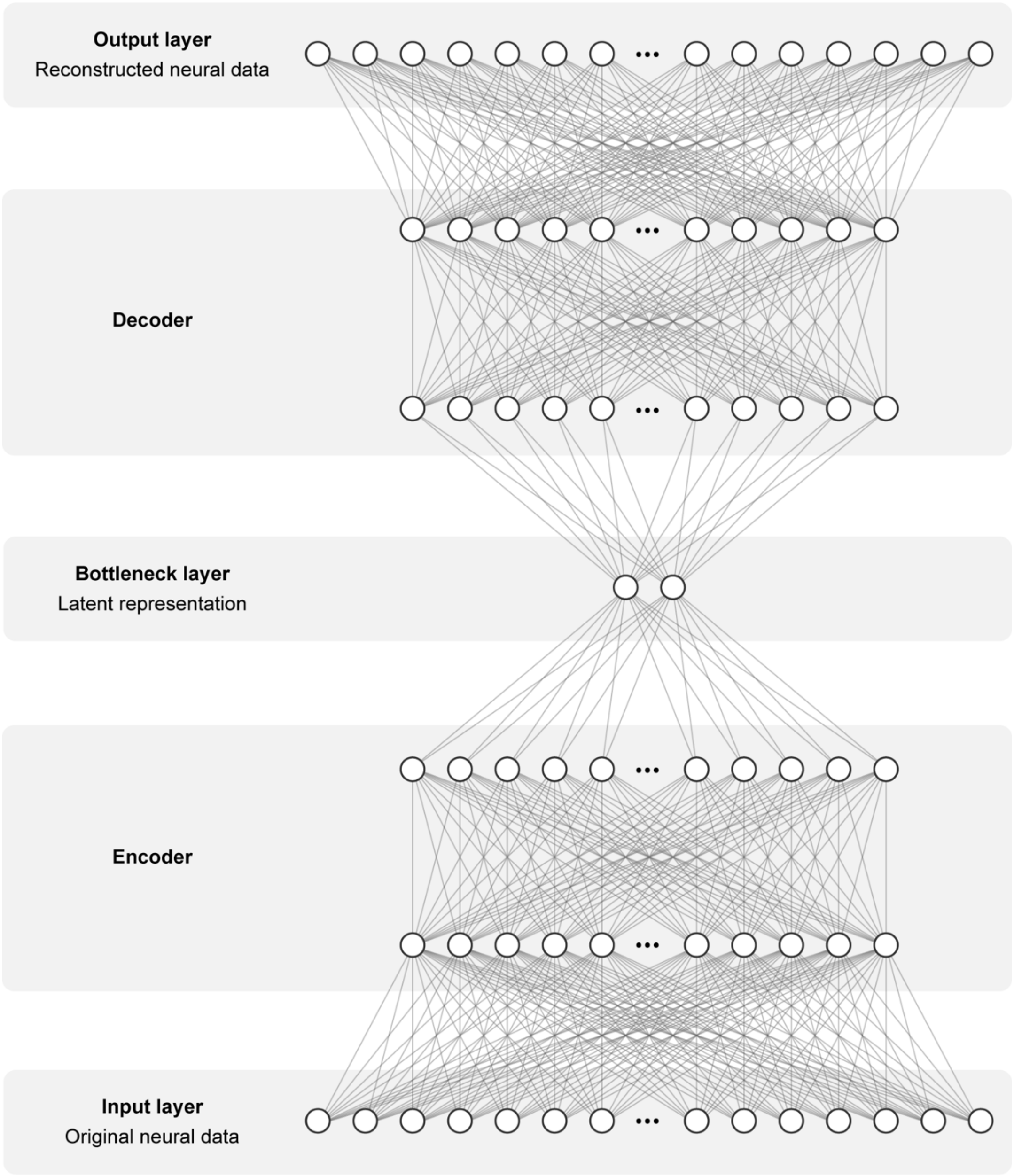

Supplementary Fig. 8 | Illustration of the autoencoder used for latent representation analyses.

The autoencoder included an input layer and output layer of the same size, in which the number of nodes corresponded to the animal's number of neurons multiplied by 10 (as activity was binned in consecutive 10 ms bins). Activity passed through the encoder and decoder components (each including two layers of 500 nodes) and a "bottleneck" layer with 2 nodes whose activity was used to define the two-dimensional latent projection space used for this analysis.

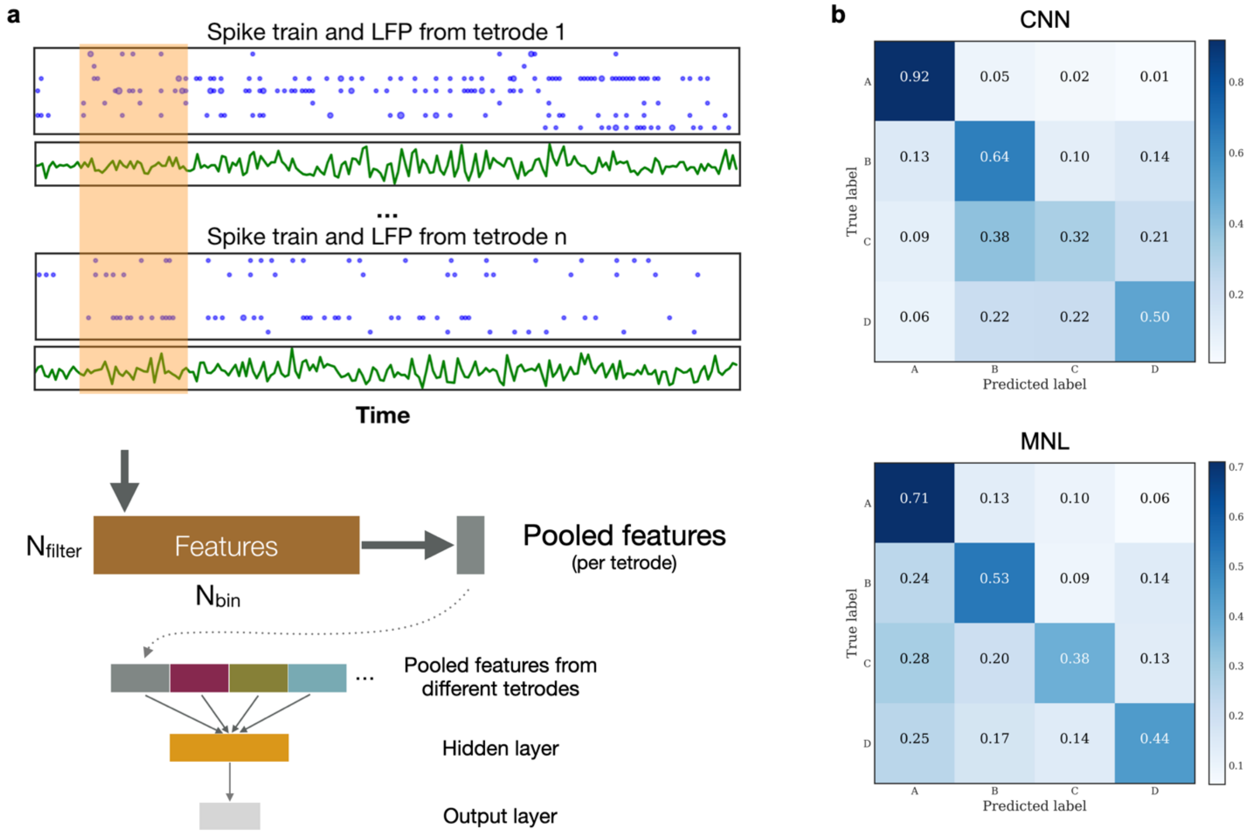

Supplementary Fig. 9 | Illustration of the convolutional neural network (CNN) used for the neural decoding analyses, and comparison to a multinomial logistic (MNL) regression model.

a Neural activity from each trial (-2 s to 2 s relative to port entry) was segmented into 250 ms time windows. The model mapped the LFP and spike activity data within the time window to a hidden layer vector and then made a prediction based on that vector. More specifically, the neural network performed convolution on each tetrode separately. The LFP and spike activity obtained from each tetrode were combined into one multivariate time series passing through filters to produce convolution features. Next, time-averaged features from all the tetrodes were concatenated together and passed into a hidden layer before output prediction. For each trial, the 250-ms time window starting from 150 ms after port entry was used to train the decoding model. For training the neural network, we used a variant of stochastic gradient descent algorithm and followed the early stopping rule (see methods). **b** Confusion matrices for the CNN (top) and MNL with LASSO penalty (bottom). Each confusion matrix shows the proportions of correct and incorrect model classifications for each odor. For this comparison, the CNN and MNL models were both trained on the same window (150-400 ms relative to port entry) for all subjects with 10-fold cross-validation. The results shown were calculated using model predictions on the validation folds. The overall classification accuracy across all four odors and five subjects of the CNN model was 64.5% while the LASSO logistic regression model had 54.6%. The CNN had higher classification accuracy than the MNL model for all the odors except odor C. More importantly, the CNN model was able to better preserve the sequential pattern of odors whereas the MNL model mis-classified many trials to odor A (as seen in the first column).

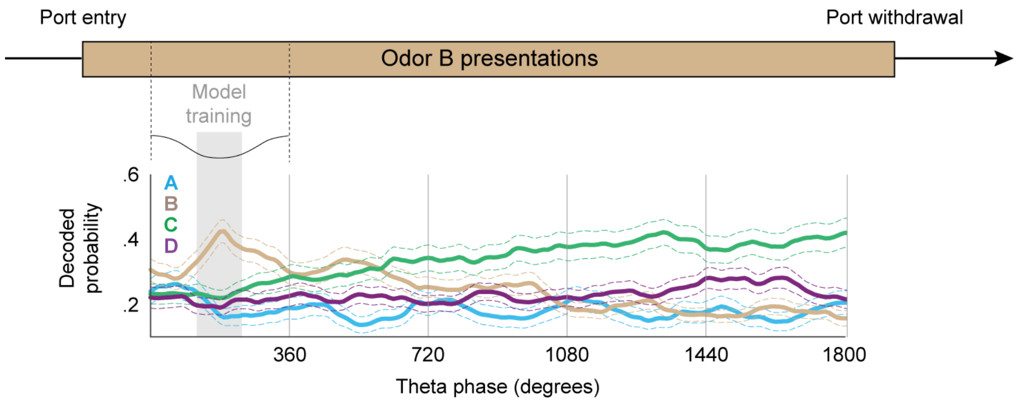

Supplementary Fig. 10 | Neural decoding of reactivation of discontiguous event sequences using the multinomial logistic regression model.

Despite overall weaker odor decoding than the CNN model, the logistic regression model shows a similar pattern of sequential activation of upcoming stimuli (odor A=sky blue, B=brown, C=green, D=purple; compare with Fig. 4c). The model was trained using neural activity during the trough of the first theta cycle starting 100 ms after port entry, and tested across all phases shown. Data depict mean ± 95% multinomial CI.

Subjects		InSeq trials					OutSeq trials				Bayesian model (Fig. 2)	Autoencoder (Fig. 3)	CNN model (Fig. 4)	Logistic regression (Fig. 5-6)
Rat ID	Rat Name	Odor A	Odor B	Odor C	Odor D	*Odor E*	Pos 2	Pos 3	Pos 4	*Pos 5*	InSeq correct trials (A-E)	All InSeq (A-D) and OutSeq (Pos 2-4) trials	All InSeq correct trials (A-D)	As Fig. 4, but excluding 20% trials with lowest theta ampl.
1	Barat	45 (1)	34 (0)	25 (0)	26 (0)	*21 (0)*	2 (1)	5 (5)	1 (2)	*3 (2)*	60	150	153	122
2	Buchanan	68 (8)	38 (5)	26 (4)	42 (1)	*29 (5)*	8 (6)	8 (3)	6 (0)	*7 (6)*	35	223	229	183
3	Mitt	66 (13)	57 (2)	47 (3)	37 (4)	*23 (10)*	3 (1)	5 (3)	4 (6)	*4 (4)*	70	251	252	201
4	Stella	53 (5)	40 (5)	30 (3)	29 (3)	*24 (2)*	4 (0)	8 (3)	6 (0)	*5 (2)*	60	189	194	155
5	SuperChris	55 (1)	41 (9)	37 (2)	31 (3)	*26 (5)*	7 (0)	8 (0)	6 (3)	*5 (1)*	50	203	206	164
Total		290 (28)	210 (21)	165 (12)	165 (11)	*123 (22)*	24 (8)	34 (14)	23 (11)	*24 (15)*	275	1016	1034	825

Supplementary Table 1 | Trial counts and inclusion criteria for each model.

Trial counts for correct InSeq and OutSeq trials (incorrect trials shown in parentheses) and subset of trials used in each model. For the Bayesian reconstructed time model (Fig. 2), we only included trials from full sequences in which all items (A-E) were presented InSeq and were correctly identified, to balance the number of trials in temporal coding plots across odor type. The autoencoder (Fig. 3) included all InSeq trials (Odors A-D; correct and incorrect) and all OutSeq trials in sequence position 2-4 (correct and incorrect). The CNN model (Fig. 4) included all InSeq correct trials of Odors A-D. The logistic regression model (Fig. 5-6) included the same trials as the CNN model but excluded the 20% of trials with the lowest theta amplitude. Data from only one session were used for each subject. Odor E trials (and OutSeq trials in the last sequence position) are italicized to indicate their exclusion from a number of analyses due to reduced sampling and decoding accuracy (< 0.15 relative to > 0.32 for other odors; see diagonal in Fig. S9b).

Subjects			Bayesian model (Fig. 2b)	Bayesian model (Fig. 2e)	Autoencoder (Fig. 3)	CNN model (Fig. 4)	Logistic regression (Fig. 5-6)
Rat ID	Rat Name	All Neurons	Neurons active during subset of InSeq trials (A-E)	Neurons active during full sequence (A-E)	All neurons	All neurons	Neurons active during InSeq correct trials (A-D; 8 theta cycles)
1	Barat	92	50	82	92	92	83
2	Buchanan	79	38	78	79	79	79
3	Mitt	104	64	101	104	104	100
4	Stella	49	26	47	49	49	49
5	SuperChris	46	39	46	46	46	46
Total		370	217	354	370	370	357

Supplementary Table 2 | Neuron counts and inclusion criteria for each model.

The autoencoder (Fig. 3) and CNN model (Fig. 4) included all neurons. The Bayesian reconstructed time model (Fig. 2) and logistic regression model (Fig. 5-6) only included active neurons (neurons that fired at least one spike) during included trials (see Supplementary Table 1), though the logistic regression model considers a longer trial period (a total of 8 theta cycles: 3 before port entry and 5 cycles after). Data from only one session were used for each subject.

Time window	Trial type	One-way ANOVAs			Linear trends				
		MS	F value	*p* value	Slope	R^2 (means)	R^2 (effect size)	F value	*p* value
0:250 ms relative to port entry	InSeq	11.55	10.68	$p < 0.0001$	0.1176	0.996	0.01189	31.92	$p < 0.0001$
	OutSeq	93.04	8.589	$p < 0.0001$	1.473	0.9908	0.1827	25.53	$p < 0.0001$
250:500 ms relative to port entry	InSeq	269.2	46.51	$p < 0.0001$	0.564	0.9817	0.04907	137	$p < 0.0001$
	OutSeq	3974	5.142	$p = 0.0023$	9.428	0.9503	0.1133	14.66	$p = 0.0002$
-250:0ms relative to port withdrawal	InSeq	475.8	60.21	$p < 0.0001$	0.7224	0.9106	0.05815	164.5	$p < 0.0001$
	OutSeq	617.5	5.237	$p = 0.0020$	3.805	0.9962	0.1207	5.237	$p = 0.002$
0:250 ms relative to port withdrawal	InSeq	40.15	14.47	$p < 0.0001$	0.2182	0.9846	0.01588	42.75	$p < 0.0001$
	OutSeq	552.6	3.055	$p = 0.0313$	2.68	0.552	0.04108	5.059	$p = 0.0264$

Supplementary Table 3 | ANOVA results for autoencoder lag analysis (Fig. 3b).

Detailed report of one-way ANOVAs and linear trends analyses. Degrees of freedom were [3, 2652] for InSeq and [3, 114] for OutSeq analyses.
