# Supplementary material for: Hippocampal ensembles represent sequential relationships among an extended sequence of nonspatial events
Source: Nat Commun. 2022 Feb 8;13:787. doi: 10.1038/s41467-022-28057-6 (PMC8825855; doi:10.1038/s41467-022-28057-6)
Supplement: Supplementary file 3 — Reporting Summary [file 41467_2022_28057_MOESM3_ESM.pdf]

nature research

Corresponding author(s): Norbert J. Fortin
Last updated by author(s): November 2, 2021

Reporting Summary

Nature Research wishes to improve the reproducibility of the work that we publish. This form provides structure for consistency and transparency in reporting. For further information on Nature Research policies, see Authors & Referees and the Editorial Policy Checklist.

Statistics

For all statistical analyses, confirm that the following items are present in the figure legend, table legend, main text, or Methods section.

n/a	Confirmed	
☐	☒	The exact sample size (*n*) for each experimental group/condition, given as a discrete number and unit of measurement
☒	☐	A statement on whether measurements were taken from distinct samples or whether the same sample was measured repeatedly
☐	☒	The statistical test(s) used AND whether they are one- or two-sided<br>*Only common tests should be described solely by name; describe more complex techniques in the Methods section.*
☒	☐	A description of all covariates tested
☐	☒	A description of any assumptions or corrections, such as tests of normality and adjustment for multiple comparisons
☐	☒	A full description of the statistical parameters including central tendency (e.g. means) or other basic estimates (e.g. regression coefficient) AND variation (e.g. standard deviation) or associated estimates of uncertainty (e.g. confidence intervals)
☐	☒	For null hypothesis testing, the test statistic (e.g. *F*, *t*, *r*) with confidence intervals, effect sizes, degrees of freedom and *P* value noted<br>*Give P values as exact values whenever suitable.*
☐	☒	For Bayesian analysis, information on the choice of priors and Markov chain Monte Carlo settings
☒	☐	For hierarchical and complex designs, identification of the appropriate level for tests and full reporting of outcomes
☒	☐	Estimates of effect sizes (e.g. Cohen's *d*, Pearson's *r*), indicating how they were calculated

Our web collection on statistics for biologists contains articles on many of the points above.

Software and code

Policy information about availability of computer code

Data collection	The electrophysiological and behavioral data were recorded using a multichannel acquisition processor (Plexon Inc).
Data analysis	Data pre-processing (e.g., sorting individual units) was performed using Offline Sorter (Plexon Inc). Custom scripts (Matlab or Python) were used for data analysis and figure generation, which are available at https://doi.org/10.5281/zenodo.5579785.

For manuscripts utilizing custom algorithms or software that are central to the research but not yet described in published literature, software must be made available to editors/reviewers. We strongly encourage code deposition in a community repository (e.g. GitHub). See the Nature Research guidelines for submitting code & software for further information.

Data

Policy information about availability of data

All manuscripts must include a data availability statement. This statement should provide the following information, where applicable:

- Accession codes, unique identifiers, or web links for publicly available datasets
- A list of figures that have associated raw data
- A description of any restrictions on data availability

The data used in this study have been deposited at https://doi.org/10.7280/D14X30.

Field-specific reporting

Please select the one below that is the best fit for your research. If you are not sure, read the appropriate sections before making your selection.

☒ Life sciences ☐ Behavioural & social sciences ☐ Ecological, evolutionary & environmental sciences

Life sciences study design

All studies must disclose on these points even when the disclosure is negative.

Sample size	Samples sizes were determined using standards in behavioral electrophysiology experiments. The dataset included 370 neurons recorded from 5 rats (range: 46-104 neurons per animal) over a minimum of 100 trials.
Data exclusions	All the data were used whenever possible. Data were only excluded when it was important to keep the sampling balanced across conditions to avoid biasing the analyses. Our rigorous approach to data exclusion is detailed in the "Data inclusion, sampling, and pooling" section of the methods and in Supplementary Figures 1-3.
Replication	Given the technical nature of this research, it is not practical to reproduce the findings in an independent cohort (as it takes several months to obtain data from one subject). Instead, we have run the analyses multiple times using different subsets of the data and different parameters and those analyses consistently confirmed the findings.
Randomization	Animals were randomly selected and all animals were included in the experimental group.
Blinding	All data acquisition was automated. All data pre-processing (e.g., cell sorting) was performed blind to the animal's behavioral performance and to the result of the analyses. Blinding to group allocation was not possible, as only one experimental group was used.

Reporting for specific materials, systems and methods

We require information from authors about some types of materials, experimental systems and methods used in many studies. Here, indicate whether each material, system or method listed is relevant to your study. If you are not sure if a list item applies to your research, read the appropriate section before selecting a response.

Materials & experimental systems

n/a	Involved in the study	
☒	☐	Antibodies
☒	☐	Eukaryotic cell lines
☒	☐	Palaeontology
☐	☒	Animals and other organisms
☒	☐	Human research participants
☒	☐	Clinical data

Methods

n/a	Involved in the study	
☒	☐	ChIP-seq
☒	☐	Flow cytometry
☒	☐	MRI-based neuroimaging

Animals and other organisms

Policy information about studies involving animals; ARRIVE guidelines recommended for reporting animal research

Laboratory animals	The animals' species (rat), strain (Long-Evans), and sex (males) was reported in the text. As is customary in the field, the animals' weight at the beginning of training was used as a proxy for their age.
Wild animals	The study did not involve wild animals.
Field-collected samples	The study did not involve samples collected from the field.
Ethics oversight	All experimental work was approved by IACUC and consistent with their recommendations.

Note that full information on the approval of the study protocol must also be provided in the manuscript.
